# Fungal empyema in complicated chronic pancreatitis: A rare possibility

**DOI:** 10.1016/j.ijscr.2019.02.040

**Published:** 2019-03-07

**Authors:** Yasir Khan, Munawar Hussain, Syed Shahabuddin

**Affiliations:** Section of Cardiothoracic, Department of Surgery, Aga Khan University Hospital, Karachi, Pakistan

**Keywords:** Fungal empyema, Chronic pancreatitis, Cystgastrostomy, Amylase rich fluid

## Abstract

**Introduction:**

Fungal empyema a rare cause of empyema thoracis is commonly associated with nosocomial infection or gastrointestinal disease with very high mortality. Its association with chronic pancreatitis is rarely been described.

**Presentation of case:**

We present a case of young male known alcoholic with chronic pancreatitis and cystgastrostomy who presented with dyspnea and fever. Thorough workup showed left sided amylase rich loculated pleural effusion. Culture grew candida albicans. Esophagogram carried out for any esophageal rupture turned out to be negative. Histopathology was negative for malignancy. Intraoperative left sided yellowish colour fluid was drained, Loculation broken and rind removed. No pleuro-peritoneal fistula could be identified. Postoperatively patient remained well and discharged on antifungal.

**Conclusion:**

No obvious cause of fungal empyema could be found except for pancreatico-pleural spread as suggested by amylase rich pleural effusion along with growth of candida spp. Hence in patient with chronic pancreatitis and cystgastrostomy pleural effusion should be evaluated for fungal infection to avoid morbidity and mortality and commence early treatment.

## Highlights

• Fungal Empyema is a rare cause of Empyema Thoracis.
• There is possibility of Fungal Empyema in Complicated Chronic Pancreatitis.
• Early Treatment is important to avoid morbidity and mortality.

## 1 Introduction

The following case report has been reported from Our University Hospital which is an internationally recognized teaching hospital and a tertiary care centre, in accordance with the SCARE guidelines for case reports [1]. The occurrence of fungal infection is rising rapidly worldwide in hospitalized population with high mortality [2]. Fungal empyema is rare and common causes are nosocomial or GI perforation. Common organism isolated is candida albicans [3]. Pleural effusion and pleuroperitoneal fistula (PPF) due to chronic pancreatitis has been reported [4]. However fungal empyema in non-nosocomial patient with chronic pancreatitis has not been reported.

## 2 Presentation of case

**Fig. 1:**
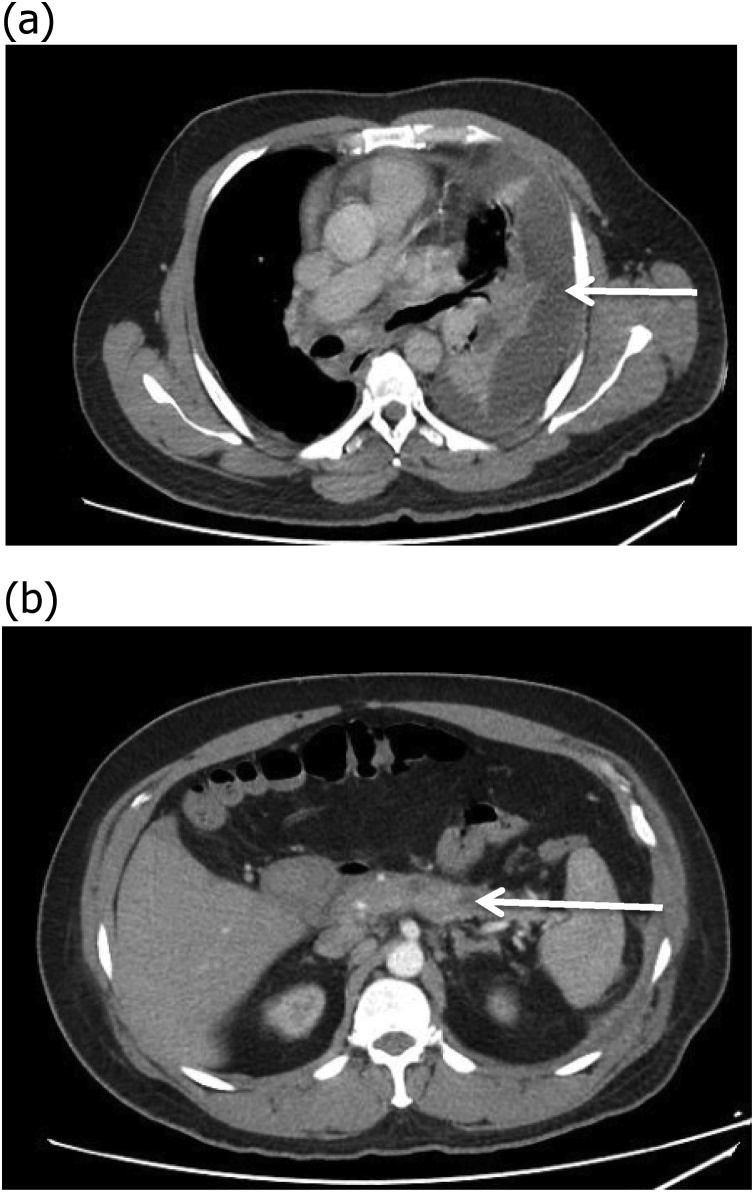
a. CT chest showing left sided loculated effusion. b. Diffuse thickening along the pancreas with atrophy of tail of pancreas and prominent pancreatic duct likely representing changes of chronic pancreatitis.

**Fig. 2:**
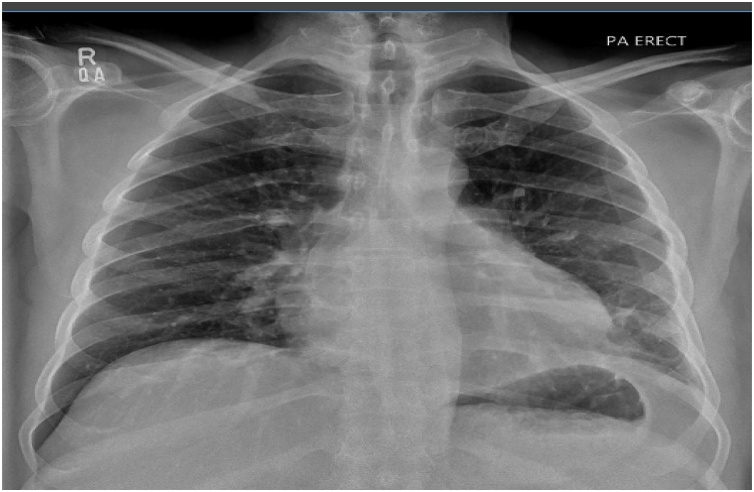
Chest X-ray six month Postoperative.

A 47 years old male known case of diabetes, ex alcoholic, chronic pancreatitis and post cystgastrostomy (6 years back) and was admitted 2 years back with small infected pseudocyst which was managed conservatively. Now patient was admitted with fever and dyspnea. Blood workup showed raised total leucocytes count with predominant neutrophils. Serum amylase and lipase were slightly elevated. Chest x-ray showed left sided effusion. Ultrasound chest with diagnostic tap revealed loculated effusion. Pleural fluid DR and Culture showed exudative neutrophilic effusion with high amylase 6000 I.U/L, and growth of candida albicans which was sensitive to fluconazole, voriconazole. Bacterial and AFB culture dint grew any organism. Pleural fluid cytology was negative for malignancy. Blood amylase level were 205 I.U/L. CT chest and abdomen with contrast showed large left sided loculated effusion (Fig. 1a) along with changes of chronic pancreatitis (Fig. 1b) and resolution of previous small pseudocyst. Images were negative for any neoplastic mass. Histopathology reported acute and chronic inflammation but negative for granulomas and malignancy. Barium swallow was carried out for any esophageal rupture as patient had high amylase in pleural effusion with candida which was unusual for community acquired patient and was reported negative. During left Video Assisted Thoracoscopy and decortication, yellowish fluid was drained, rinds were broken and removed. No fistula was appreciated in diaphragm. Postoperatively patient was started on antifungals and remained well six months postoperatively (Fig. 2).

## 3 Discussion

Couple of points has been highlighted in this case report, fungal empyema and high amylase in pleural effusion. Victoria Villena et al., reported that high amylase pleural effusion is mostly associated with malignancy, pancreatitis (especially PPF) or esophageal perforation [5]. PPF a complication of chronic pancreatitis has been reported [5]; however PPF association with fungal empyema has not been reported.

Fungal empyema thoracis is a rare form of empyema thoracis with high mortality. It is mostly found in GI perforation and nosocomial patients; rarely it is present in community acquired pneumonia [3]. Fungal empyema and high amylase in pleural effusion should raise suspicion of GI problem or malignancy in non-nosocomial patients [6]. Shiann-Chin Ko et al., reported crude mortality of 73% with fungal infections and emphasized on early treatment with drainage and systemic antifungal therapy [3].

In this case, without any history of recent hospitalization patient had fungal empyema along with amylase rich pleural effusion. Thorough investigations ruled out both malignancy and GI perforation. Since our patient had history of cystgastrostomy and readmission with infected pseudocyst which was managed conservatively, fungal empyema was thought to be due to pleuroperitoneal spread.

## 4 Conclusion

To conclude Fungal Empyema presented as a unique case requiring multidisciplinary approach. It should be included with high suspicion in patient with history of chronic pancreatitis and cystgastrostomy. It will help in early recognition with timely management, to avoid morbidity and mortality.

## Conflict of interest

None.

## Funding

None.

## Ethical approval

Ethical review exemption is in process and will update as early.

## Consent

Consent has been taken from patient for this Written informed consent was obtained from the patient for publication of this case report and accompanying images. A copy of the written consent will be made available for review by the Editor-in-Chief of this journal on request along with images.

## Author contribution

• Yasir Khan – Study Concept, Study Design, Data Collection, Manuscript Writing.
• Munawar Hussain – Critical Review of Literature, Data Interpretation, Proof reading.
• Syed Shahab– Data Analysis, Interpretation, Manuscript Drafting.

## Registration of research studies

Not Required.

## Guarantor

Syed Shahab

Assistant Professor Cardiothoracic Surgery

Aga Khan University Hospital Karachi, Pakistan.

## Provenance and peer review

Not commissioned externally peer reviewed.
